# The Effect of Perioperative Ischemia and Reperfusion on Multiorgan Dysfunction following Abdominal Aortic Aneurysm Repair

**DOI:** 10.1155/2015/598980

**Published:** 2015-12-21

**Authors:** Konstantina Katseni, Athanasios Chalkias, Thomas Kotsis, Nikolaos Dafnios, Vassilis Arapoglou, Georgios Kaparos, Emmanuel Logothetis, Nicoletta Iacovidou, Eleni Karvouni, Konstantinos Katsenis

**Affiliations:** ^1^Vascular Surgery Unit, 2nd Surgical Department, Medical School, National and Kapodistrian University of Athens, Athens, Greece; ^2^MSc “Cardiopulmonary Resuscitation”, Medical School, National and Kapodistrian University of Athens, Athens, Greece; ^3^Hellenic Society of Cardiopulmonary Resuscitation, Athens, Greece; ^4^Department of Anesthesiology, Tzaneio Hospital of Piraeus, Piraeus, Greece; ^5^Department of Biopathology, Aretaion Hospital, Medical School, National and Kapodistrian University of Athens, Athens, Greece; ^6^Neonatal Division, Department of Pediatrics, Aretaion Hospital, Medical School, National and Kapodistrian University of Athens, Athens, Greece

## Abstract

Abdominal aortic aneurysms (AAAs) are relatively common and are potentially life-threatening medical problems. The aim of this review is to provide an overview of the effect of I/R injury on multiorgan failure following AAA repair. The PubMed, CINAHL, EMBASE, Medline, Cochrane Review, and Scopus databases were comprehensively searched for articles concerning the pathophysiology of I/R and its systemic effects. Cross-referencing was performed using the bibliographies from the articles obtained. Articles retrieved were restricted to those published in English. One of the most prominent characteristics of AAA open repair is the double physiological phenomenon of ischemia-reperfusion (I/R) that happens either at the time of clamping or following the aortic clamp removal. Ischemia-reperfusion injury causes significant pathophysiological disturbances to distant organs, increasing the possibility for postoperative multiorgan failure. Although tissue injury is mediated by diverse mechanisms, microvascular dysfunction seems to be the final outcome of I/R.

## 1. Introduction

Abdominal aortic aneurysms (AAAs) are relatively common and are potentially life-threatening medical problems. The highest prevalence of AAA >3.0 cm is 5.9% and was found in white male smokers between 50 and 79 years [1]. The incidence of ruptured AAAs (rAAAs) is 5.6–17.5 per 100,000 person-years in western countries [2–4], while the overall mortality rate of patients is approximately 80–90% [1]. Despite the improvements in surgical technique, grafts, and perioperative care during the last decades, AAA repair carries a considerable risk of morbidity and death, especially in case of rupture [5].

One of the most prominent characteristics of AAA open repair is the double physiological phenomenon of ischemia-reperfusion (I/R) that happens either at the time of clamping and following the aortic clamp removal or earlier in case of rAAA [6]. In both cases, significant controversy exists over which tissues are most susceptible to periods of I/R. Although the two main targets of I/R seem to be the gastrointestinal tract and the muscle mass of the lower-limbs, much of the data so far appears conflicting. Nevertheless, this “two-hit” I/R is responsible for the extensive systemic inflammatory response and the increased incidence of the postoperative multiorgan failure (MOF) [7]. The aim of this narrative review is to provide an overview of the effect of I/R injury on MOF following AAA repair.

## 2. Methods

The PubMed, CINAHL, EMBASE, Medline, Cochrane Review, and Scopus databases were comprehensively searched for relevant articles, using keywords such as “aortic aneurysm,” “abdominal,” “ischemia,” “reperfusion injury,” “inflammation,” “brain,” “heart,” “lung,” “liver,” “renal,” and “multiple organ failure.” All original papers and reviews of relevance to the particular question were included after thorough examination, while cross-referencing was performed using the bibliographies from the articles obtained. Articles retrieved were restricted to those published in English. Articles were selected for inclusion if they concerned the pathophysiology of I/R and the systemic effects of I/R.

## 3. Abdominal Aortic Cross-Clamping

Clamping the thoracic or abdominal aorta causes rapid and important hemodynamic, cellular, and molecular changes that can result in major complications in several organ systems.

### 3.1. Major Pathophysiological Disorders

Aortic cross-clamping results in an immediate decrease in blood flow to the distal tissues, while it rapidly increases mean arterial pressure (MAP) due to an increase in afterload [8]. Although the effect of aortic cross-clamping on cardiac output varies, blood flow distal to the clamp is maintained due to the increased proximal MAP [9].

One of the most significant manifestations of clamping is the release of catecholamines, which increases systemic vasospasm and results in venoconstriction and decrease in venous capacity [10]. This is of particular importance and may affect venous return in infraceliac clamping, as the change in preload depends upon the tone of the splanchnic veins in this case; in patients with high splanchnic vascular tone venous return to the heart increases, while when splanchnic venous tone is low, preload decreases due to venous blood pooling. Of note, pulmonary vascular resistance also increases after the clamping due to the increase in catecholamines as well as due to the retrograde loading of the aorta, which may result in ventilation-perfusion mismatch, pulmonary hypertension, and myocardial dysfunction.

**Figure 1:**
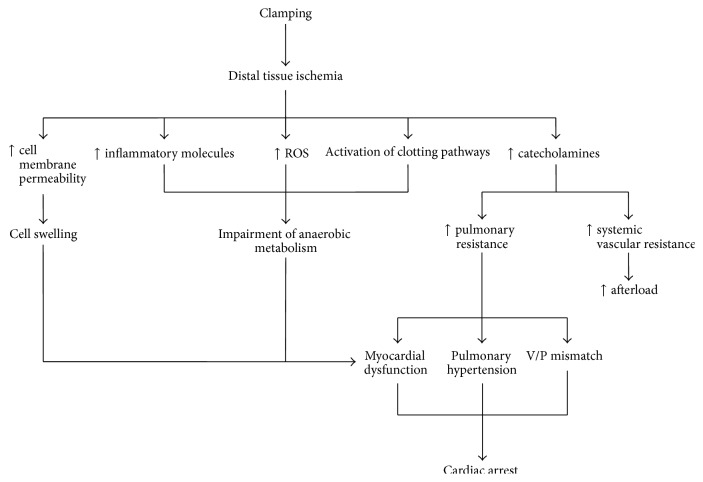
Pathophysiological manifestations of aortic clamping resulting in hemodynamic compromisation. ROS: reactive oxygen species.

Due to the rapid decrease in blood flow, oxygen consumption in tissues distal to the clamp decreases and oxygen uptake in tissues above the clamp increases, possibly due to sympathetic-induced vasoconstriction and reduced blood flow in arterioles and capillaries [11]. Cell membrane permeability increases due to anaerobic cellular metabolism, lactic acidosis, decreased glycogen, and low adenosine triphosphate, leading to cellular swelling [6]. Cellular metabolism is further impaired due to the upregulation of several other inflammatory molecules and oxygen radicals and the activation complement and clotting pathways (Figure 1).

### 3.2. Abdominal Visceral Effects

**Figure 2:**
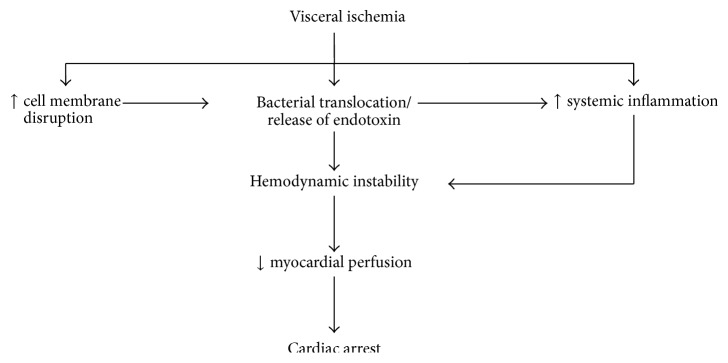
Effect of ischemia-induced bacterial translocation on systemic inflammation in patients with abdominal aortic aneurysm.

Visceral ischemia develops in 1–10% of patients undergoing aortic surgery and is characterized by high mortality [10]. Immediately after the clamping, visceral tissues suffer an acute hypoxic insult due to the sudden decrease in celiac, superior mesenteric, or inferior mesenteric blood supply. The degree of hypoxia is depending not only on the collateral circulation but also on several other intraoperative factors, such as hypotension, hypovolemia, thrombosis, microembolism, or intestinal artery lesions [11, 12]. Visceral ischemia increases intestinal permeability and enhances bacterial translocation and systemic inflammation, which may complicate the postoperative period. Of note, bacterial translocation has been implicated as a potent cause for cardiac arrest, especially in case of endotoxin-release species [13]. In a recent study, over one-third of out-of-hospital adults were bacteremic upon presentation [14]. These patients have greater hemodynamic instability and significantly increased short-term mortality. In another recent study of patients who suffered in-hospital cardiac arrest, the incidence of preexisting pneumonia was 12.1% [15]. In this study, a subset of these patients developed abrupt cardiac arrest without signs of hypotension, overt shock, respiratory failure, or severe metabolic derangements (Figure 2).

### 3.3. Renal Effects

Renal perfusion is affected by the level of cross-clamping; it decreases by 80% and 45% during suprarenal and infrarenal aortic cross-clamping, respectively. The sudden decrease in renal perfusion increases the levels of angiotensin II that, in turn, increases renal vesicular resistance by 70% shortly after the clamping, redistributing blood flow away from the renal medulla and enhancing renal hypoperfusion [10]. This change further enhances the release of angiotensin II, creating a vicious circle, which together with the lower-limb ischemia and the resultant muscle necrosis and myoglobinuria may have disastrous consequences on renal tissue [16].

## 4. Reperfusion

Reperfusion happens either following the aortic clamp removal or earlier in patients with rAAA and is paradoxically responsible for additional injurious events after the insult of ischemia [6]. In fact, the reperfusion damage may exceed the original ischemic injury, indicating the need for a better understanding of the underlying pathophysiology in patients with AAA. The primary hemodynamic response to unclamping of the aorta is significant hypotension due to decreased afterload, hypoxia-induced peripheral vasodilation, and a marked release of vasodilatory and myocardial-depressant metabolites from areas distal to the clamp [17].

### 4.1. Immediate Inflammatory Responses

After restoration of blood flow, the body is characterized by widespread activation of several inflammatory pathways and biochemical changes. Reoxygenation enhances the production of oxygen radicals (ROS), which are implicated in lipid peroxidation, complement activation, platelet aggregation, and white cell activation. As a result, microvascular dysfunction will occur not only in the reperfused tissues but in distal organs as well. The distant organ injury has been shown to be neutrophil dependent and this appears to be due to the activation of resident neutrophils in these organs rather than the influx of neutrophils from the site of injury [18, 19]. However, resident neutrophils can be activated by several molecules generated at the site of surgical injury which enter the systemic circulation [20].

### 4.2. Polymorphonuclear Neutrophils

An abundance of evidence indicates the central role of polymorphonuclear neutrophils in the pathophysiology of I/R [21–26]. The emigration of polymorphonuclear neutrophils from the postcapillary venules to the area of inflammation is mediated by the upregulation of adhesion molecules, several chemoattractants, chemokines, and integrins [27]. The confluent polymorphonuclear neutrophils can damage the tissues in several ways including secretion of proteolytic enzymes, production of free radicals, and impairing the microcirculation [28, 29].

### 4.3. Reactive Oxygen Species

The increase of ROS has been documented shortly after the beginning of AAA repair. Thompson et al. reported that the production of ROS was maximal after 5 min of lower-limb reperfusion but declined after 30 min of reperfusion [30]. It was also reported that rupture patients had raised F(2)-isoprostane level on arrival at hospital, indicating that they were exposed to an early oxidative injury, strengthening the “two-hit” hypothesis [31]. Oxygen radicals may also suppress adenosine triphosphate synthesis, inactivate the metabolic enzymes and exacerbate the depletion of antioxidant reserves, disrupt cellular and mitochondrial membrane, impair the function of pumps and cause severe derangements of intracellular electrolytes, impair b-adrenergic signaling, intensify endothelial injury, and enhance apoptosis [21].

### 4.4. Nitric Oxide

The role of nitric oxide (NO) in I/R seems to be dichotomous as it has both cytotoxic and cytoprotective effects. Nitric oxide has many beneficial effects such as scavenging of oxygen free radicals, maintenance of normal vascular permeability, inhibition of smooth muscle proliferation, reduction of PMN adherence, and inhibition of platelet aggregation [32–35]. On the other hand, large amounts of NO have been implicated in tissue injury, bacterial translocation, mucosal apoptosis, and pulmonary injury [36–40].

### 4.5. Complement

**Figure 3:**
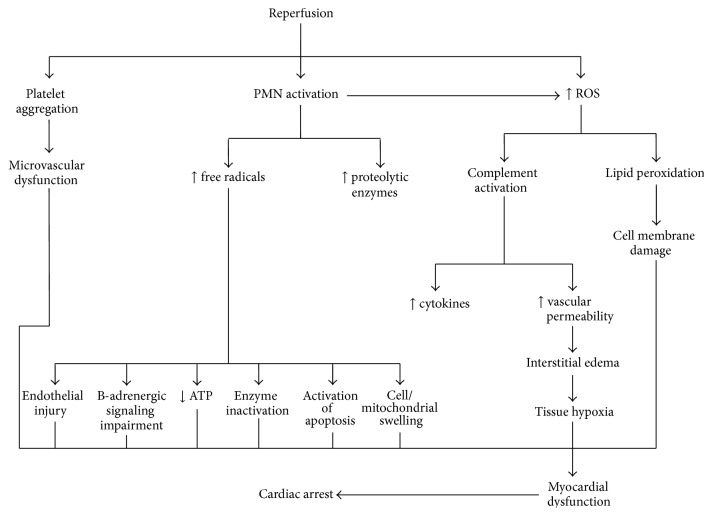
Late reperfusion-induced pathophysiological disorders that lead to cardiac arrest. PMN: polymorphonuclear leukocytes; ROS: reactive oxygen species; ATP: adenosine triphosphate.

Several studies suggested that complement system has a major role in the pathogenesis of I/R [41–43]. Its activation leads to a series of proinflammatory events including upregulation of cytokines such as TNF-*α* and interleukin IL-1 [43], increasing vascular permeability, and enhancing the formation of ROS. This leads to cell membrane damage and cellular and mitochondrial swelling, which favors the formation of tissue edema that expands the interstitium of the injured organ and increases the diffusion distance for the already diminished oxygen [21]. Of note, the oxidative stress and ROS formation reach their peak during the ischemic attack (15–60 min of clamping), while PMN infiltration is maximal during reperfusion (Figure 3) [5].

### 4.6. Interleukins

Abdominal aortic aneurysm repair was associated with an increase in the levels of several cytokines. IL-6 was shown to increase in plasma during AAA repair and remains elevated during the postoperative period [44–52]. As for the TNF-*α*, however, the results have been conflicting with some authors showing no change in TNF-*α* levels [45, 46, 53] and others demonstrating a rise [44, 50]. Furthermore, there is evidence that the level of clamping is related to the increase in TNF-*α*, while although there was no measurable change in TNF-*α* levels in AAA patients with infrarenal clamps, patients with suprarenal clamps did have a small but significant increase in TNF-*α* [52–54]. On the other hand, IL-8 has been less commonly measured but does appear to increase during and shortly after AAA repair [6].

### 4.7. Coagulation and Fibrinolysis

Although several changes in the coagulation cascade were demonstrated to occur as a result of AAA repair, it remains unknown whether these are predominantly procoagulant or fibrinolytic. Novelli et al. demonstrated a procoagulant state in patients undergoing repair of elective infrarenal AAA [54], which has been also demonstrated in animal and human studies [55, 56]. On the contrary, Wijnen et al. reported that patients undergoing supracoeliac clamping for thoracoabdominal aneurysm repair developed a profibrinolytic state within 20 min of aortic clamping [57]. Collectively, although the resulting state may be dependent on the severity and/or size of the aneurysm, these findings warrant further research.

## 5. Postoperative Effects of Ischemia/Reperfusion Injury

### 5.1. Intestine

Among all, the intestine seems to be the most sensitive organ to I/R injury due to the presence of labile cells which are very susceptible to ischemia [58]. Of note, these cells are located at the tips of the villi, a place that is supplied by the end of the distribution of a central arteriole, thus increasing their vulnerability to ischemia compared to cells located within the crypts [6]. Nevertheless, the effects of I/R on the gastrointestinal tract have a significant impact on the postoperative physiology of the human body and have received considerable interest during the last years.

Research so far has shown that the main mechanisms by which the intestine enhances the postoperative inflammatory response are the ischemic insult, intraoperative bowel manipulation and mesenteric traction, intestinal hypothermia, release of vasoactive mediators from the intestinal endothelium, impaired mucosal permeability, and endotoxin translocation [6].

After AAA repair, the intestinal mucosa produces various acute-phase proteins, hydrogen peroxide, hormones, and cytokines [59–61]. These, together with ROS formation, complement activation, and PMN infiltration, are deleterious to the intestinal and distal organ microvasculature, altering the absorptive function of the intestine and leading to bowel infarction, short-bowel syndrome, systemic inflammatory response syndrome, acute respiratory distress syndrome, and MOF [62]. Another study of 21 patients undergoing open AAA repair showed that ischemia-induced acidosis of the sigmoid colon was positively correlated with the concentration of TNF-*α* and IL-6 [63]. In two other studies, high levels of IL-6 were found in inferior mesenteric vein and portal vein compared to systemic blood during aortic clamping and reperfusion after AAA repair [64]. Furthermore, in a study of thoracoabdominal aneurysm repair, the levels of plasma TNF-*α* and IL-10 were positively correlated with visceral ischemic time [17, 53]. Visceral ischemia may also result in the development of coagulopathy from increased intestinal permeability and bacterial translocation or from hepatic ischemia and primary fibrinolysis [17].

Several human and animal studies have demonstrated the presence of endotoxin in systemic circulation both after AAA rupture and aortic clamping mainly due to impaired mucosal barrier [63, 65–68]. However, the effect of laparotomy on endotoxin levels remains unknown despite the evidence supporting its role in endotoxin translocation [67]. Endotoxin has been identified as major activator of the inflammatory cascade after AAA repair; endotoxemia is known to induce and/or enhance PMN and complement activation, cytokine release, mucosal permeability, and clotting pathways [6, 62].

### 5.2. Distal Vasculature

The vasculature is one of the most vulnerable targets of I/R after AAA repair. After I/R, the main features of the impaired vessels are the loss of normal endothelial function and disturbances of vascular resistance [20]. Several cytokines exert profound effects on vascular tone resulting in hemodynamic disturbances and reduced organ perfusion [51], while the damaged endothelium produces prostaglandins and thromboxanes and upregulates the expression of adhesive molecules which promote PMN activation [69].

Despite the fact that the NO pathway may be severely affected after I/R, different cytokines can have different effects on nitric oxide synthase (NOS) [70]. It has been demonstrated that TNF-*α* and IL-1*β* increase NO production, enhancing systemic hypotension and decreasing the response to exogenous vasoconstrictors [20]. In addition, this changes the site of NO production from endothelial cells to smooth muscle cells, increasing the expression of adhesion molecules and PMN infiltration. Interestingly, the effects of TNF-*α* and IL-1*β* can cause further cytokine release from the endothelial and vascular smooth muscle cells, further increasing the severity of inflammation [71].

Endothelins (ETs) is a family of potent vasoconstrictors produced by the injured vascular endothelium, acting through stimulation of the ET-A and ET-B receptors of vascular smooth muscle cells [72]. Stimulation of ET-A causes vasoconstriction, while the ET-B receptors include two subtypes, ET-B1 and ET-B2, which mediate vasodilation and vasoconstriction, respectively. Until now, three isoforms have been identified, with ET-1 being the most powerful vasoconstrictor [73]. In patients with AAA repair, the ETs can increase PMN adhesion and mucosal secretions, while they decrease tissue blood flow and ATP [74].

### 5.3. Kidneys

Even after the aorta is unclamped and normal hemodynamics are restored, renal blood flow and GFR may remain low for some time [17]. Acute renal failure may occur as a result of aortic clamping or I/R injury, but it may represent also an exacerbation of preexisting chronic renal failure. Animal studies have demonstrated elevated level of various cytokines, such as IL-1, IL-2, IL-6, TNF-*α*, and *β*, and interferon after renal I/R [20]. The injured glomerular cells respond by producing ROS, complement, NO, and other cytokines, while the mesangial cells produce IL-1, IL-6, and IL-8, aggravating local inflammation, renal failure, and MOF [75].

### 5.4. Heart

Patients with AAA are at higher risk of cardiovascular complications, especially ischemic heart disease following surgery. The risk for postoperative cardiac complications is maximal in patients with prior history of coronary artery disease, heart attack, or heart failure [20], with the major pathophysiological mechanism being the enhanced inflammatory response after I/R. The increase in cytokines, such as IL-2, IL-1*β*, IL-6, IFN-*γ*, and TNF-*α*, worsens cardiac function through direct and indirect mechanisms. Although the first are not clear, the later include the stimulation of iNOS expression which increases local NO concentration, leading to impaired adrenergic and cholinergic stimulation [76]. This reduces ventricular compliance, thus decreasing cardiac output and coronary perfusion [21, 77, 78].

During reperfusion, the injured myocytes and endothelial cells generate ROS. The oxygen radicals increase membrane damage intensifying endothelial injury, which increases the exchange vessel's permeability and leads to increased microvascular filtration and myocardial edema [79]. Myocardial edema increases cardiac chamber stiffness and impairs passive relaxation further impairing cardiac function. Moreover, microvascular perfusion is compromised due to myocyte swelling, the compression of coronary microcirculation by the existing edema, and the potent administration of exogenous vasopressors. In addition, the I/R-mediated activation of blood coagulation leads to the formation of microthrombi [80], which, together with the vasopressor-induced platelet aggregation and the accumulation of activated neutrophils and platelets in microvessels [81], contribute to microvascular obstruction, decreased myocardial perfusion, and myocardial ischemia.

### 5.5. Lungs

Patients who undergo AAA repair tend to be at high risk for postoperative complications due to advanced age, chronic pulmonary disease, or smoking [17]. It has been reported that postoperative respiratory failure develops in almost 20% of patients who undergo AAA repair; pulmonary arterial pressure and pulmonary vascular resistance may increase after the aorta is unclamped, possibly because of the release of microemboli into the pulmonary circulation and the resultant release of vasoconstrictive compounds from the lungs [17].

Although respiratory failure following AAA surgery usually manifests as part of MOF [20], the circulating cytokines may* ab initio* affect the respiratory function during the postoperative period. Considering that the pulmonary vasculature acts as a neutrophil pool in normal physiological states, the lungs can be one of the major sites of increased inflammatory response following AAA surgery. The I/R induced cytokines may pass in the pulmonary circulation and activate local endothelium and the resident leucocytes which migrate from the pulmonary vasculature into the interstitial and alveolar spaces and produce several cytokines and ROS, promoting chemotaxis, local inflammation, and ARDS [82, 83]. Moreover, the anaphylatoxins C3a and C5a increase pulmonary vascular tone and pulmonary capillary permeability and activate mast cells to release histamine, causing further pulmonary injury [17]. In addition, the activated leucocytes enter the systemic circulation and together with the ARDS-induced hypoxemia contribute to distant organ injury by releasing ROS and elastase, which also increase microvascular permeability [17].

### 5.6. Spinal Cord

Perioperative spinal cord I/R is an unpredictable event. During ischemia, the symptoms may include sensory and motor deficits associated with bladder or rectal incontinence with conservation of vibratory and proprioceptive sensation. Predisposing factors include aneurysm extent, open surgical repair, prior distal aortic operations, and perioperative hypotension [84].

Although the etiology of spinal cord ischemia after AAA surgery may be multifactorial, the fundamental cause is always an alteration in blood supply to the spinal cord. Of note, hypotension plays an important role in the cause of spinal cord I/R, which may occur without occlusion of the anterior spinal artery, especially in patients with severe shock, and should be avoided during the perioperative period by augmenting spinal cord perfusion [85, 86].

## 6. Ruptured AAAs

Patients with rAAA usually present in a critical condition characterized by severe pathophysiological disorders. The principle difference between elective and rAAA repair is the period of hemorrhagic shock; preclamping hemorrhagic shock and related impaired tissue perfusion accompany the ischemia and reperfusion of the lower torso during surgical treatment of rAAA [87].

**Figure 4:**
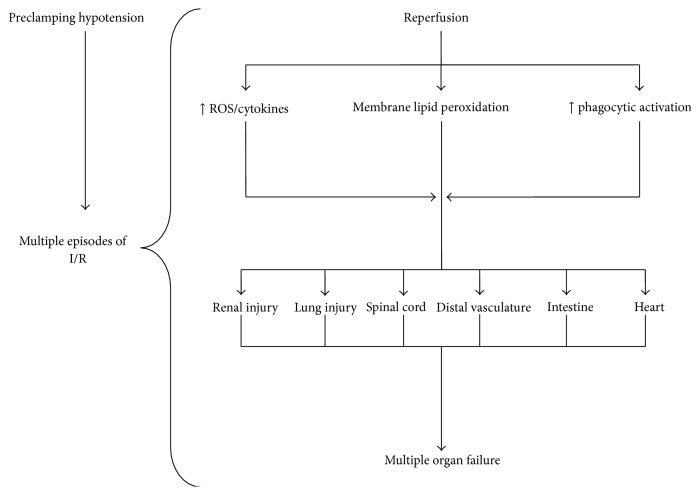
The main reperfusion-induced pathophysiological disorders that lead to distal organ injury after abdominal aortic aneurysm repair. These disorders may be significantly more severe in patients with ruptured aneurysm due to multiple episodes of ischemia-reperfusion. ROS: reactive oxygen species; I/R: ischemia-reperfusion.

Patients with rAAA may suffer multiple “two-hit” episodes, (i.e., multiple episodes of ischemia and reperfusion) prior to hospital admission due to hemodynamic fluctuations (hypotension) and the effect of compensatory mechanisms (tachycardia, changes in vascular resistance) which may enhance the inflammatory response and maximize the deleterious effects of postoperative I/R, increasing the vulnerability of several organs, especially in case of patients with preexisting diseases. This complex situation leads to systemic inflammatory response syndrome and MOF by causing local or distant organ damage such as lungs, liver, and heart. Although the existence and duration of “multiple-hit” episodes are hard to define, they depend on the severity of the injury and the physiological reserves of the patient. Therefore, older patients may suffer I/R episodes for a short period and have increased intraoperative complications, while younger individuals with high reserves may have been subjected to long periods of I/R which increases the possibility for postoperative distal organ failure [6, 10] (Figure 4).

During ischemia, the lack of oxygen leads to anaerobic metabolism and depletion of energy stores. Once the energy stores are depleted, membrane ion gradients begin to fail, membranes leak, cells swell, and the process of irreversible injury begins. Reperfusion restores tissue oxygenation but initiates an inflammatory response that has been shown to cause further tissue injury [88, 89]. Activated oxygen species and their metabolites react with unsaturated fatty acids within the phospholipid bilayer of the cell membrane, resulting in lipid peroxidation. Furthermore, in patients with rAAA, the oxidative activity of phagocytes is significantly increased compared with elective cases [31]. Rupture of an AAA results in priming of the phagocyte oxidant capacity before operative repair; phagocyte activation contributes to the high incidence of MOF and death in this patient group.

Of note, Novelli et al. demonstrated a marked procoagulant state in patients undergoing repair of ruptured infrarenal AAA [54]. In these cases, systolic arterial pressures of 50–70 mm Hg may be well tolerated for short periods, thus limiting internal bleeding and its associated loss of platelets and clotting factors [90–94]. However, the increasing cytokines may enhance renal damage [6, 62]; TNF-*α* has been reported to induce glomerular injury in animals without prior renal insult [20], as well as to be higher in shocked patients with rAAA and in those that died, indicating its potent prognostic value [6]. In an experimental rat model of rAAA, serum TNF-*α* levels were significant and suppressed by ethyl pyruvate administration [87]. Similarly, Cai et al. used ethyl pyruvate as the resuscitation fluid in hemorrhagic shock and demonstrated that TNF-*α* level was decreased and survival was increased in the rats B [95]. Also, Kung et al. created lung injury in rats by lipoteichoic acid and reported that ethyl pyruvate suppresses TNF-*α* and prevents lung injury via anti-inflammatory effect and this effect is dose-dependent [96]. In another experimental model of rAAA, Harkin demonstrated that both C5 complement and nitric oxide synthase inhibition (iNOS) provide effective protection in the serum and lung tissue by decreasing TNF-*α* [97].

## 7. Tissue Conditioning

Research so far has shown that if the blood supply to a tissue is impaired for a short time and then restored and the process is repeated more than two times, the cells are robustly protected from a final ischemic insult when the blood supply is cut off entirely and permanently (tissue conditioning). This process can be applied prior to or after the final ischemic insult and is known as ischemic preconditioning and ischemic postconditioning, respectively. However, triggering ischemic preconditioning requires direct interference with the blood vessels of the target tissues. This problem may be solved with remote preconditioning (RP), during which ischemia in one vascular bed protects tissues supplied by other vascular beds. Remote preconditioning can be achieved with readily available equipment and may be proved an extremely cost-effective means of reducing perioperative complications.

Until now, three theories have been proposed regarding the mechanism of RP [98]. The neural theory proposes that the remote organ releases endogenous substances such as adenosine and bradykinin that activate a local afferent neural pathway, activating an efferent neural pathway that triggers end organ protection. The humoral theory suggests that the remote tissue releases adenosine, bradykinin, and/or other substances into the bloodstream, which trigger the intracellular protective pathways in end organs. Finally, the inflammatory suppression theory suggests that RP suppresses inflammation and apoptosis in cells, reducing the systemic inflammatory response.

Ali et al. investigated whether RP reduces the incidence of myocardial and renal injury in patients undergoing elective open AAA repair and reported that it reduced the incidence of postoperative myocardial injury, myocardial infarction, and renal impairment [99]. In another study, Walsh et al. showed significant reductions in urinary biomarkers of renal injury; however, they reported that it had no effect on the clinical endpoints of renal impairment or major adverse cardiac events [100]. In a single-center, prospective, double-blinded, randomized, parallel-controlled trial, limb RP attenuated intestinal and pulmonary injury in patients undergoing elective open infrarenal AAA repair without any potential risk [101]. In a prospective, randomized double-blinded control trial, Murphy et al. investigated the potential for RP to attenuate renal and myocardial injury in patients undergoing elective open AAA repair and reported that it did not reduce the risk of postoperative renal failure or myocardial injury [102]. A recent systematic review and meta-analysis reported that PR did not have a significant effect on clinical endpoints (death, perioperative myocardial infarction, renal failure, stroke, mesenteric ischaemia, hospital, or critical care length of stay) [103]. However, the heterogeneity in study inclusion and exclusion criteria and in the type of preconditioning stimulus limits the potential for extrapolation in this study. In conclusion, although several studies demonstrate that PR can reduce I/R injury, further large-scale clinical studies are required to establish the role of this simple, cost-effective intervention in AAA repair.

## 8. Conclusions

Ischemia-reperfusion injury causes widespread changes in the human body following AAA repair. The evidence so far indicates that the pathways involved in I/R injury cause significant pathophysiological disturbances to distant organs, increasing the possibility for postoperative MOF. Although tissue injury is mediated by diverse mechanisms, microvascular dysfunction seems to be the final outcome of I/R. As there are crucial gaps in our knowledge, further research is needed for the full clarification of its role in AAA patients.
